# Three-Level De-Multiplexed Dual-Branch Complex Delta-Sigma Transmitter

**DOI:** 10.3390/s18020626

**Published:** 2018-02-20

**Authors:** Anis Ben Arfi, Fahmi Elsayed, Pouya M. Aflaki, Brad Morris, Fadhel M. Ghannouchi

**Affiliations:** 1 iRadio Lab, University of Calgary, Calgary, AB T2N 1N4, Canada; fmeelsay@ucalgary.ca (F.E.); mpaflaki@ucalgary.ca (P.M.A.); fghannou@ucalgary.ca (F.M.G.); 2 Ericsson Canada Inc., Mississauga, ON K2K 2V6, Canada; brad.morris@ericsson.com

**Keywords:** highly-efficient transmitter, multi-level Complex Delta-Sigma Modulator, switch mode power amplifier, dual branch amplification

## Abstract

In this paper, a dual-branch topology driven by a Delta-Sigma Modulator (DSM) with a complex quantizer, also known as the Complex Delta Sigma Modulator (CxDSM), with a 3-level quantized output signal is proposed. By de-multiplexing the 3-level Delta-Sigma-quantized signal into two bi-level streams, an efficiency enhancement over the operational frequency range is achieved. The de-multiplexed signals drive a dual-branch amplification block composed of two switch-mode back-to-back power amplifiers working at peak power. A signal processing technique known as quantization noise reduction with In-band Filtering (QNRIF) is applied to each of the de-multiplexed streams to boost the overall performances; particularly the Adjacent Channel Leakage Ratio (ACLR). After amplification, the two branches are combined using a non-isolated combiner, preserving the efficiency of the transmitter. A comprehensive study on the operation of this topology and signal characteristics used to drive the dual-branch Switch-Mode Power Amplifiers (SMPAs) was established. Moreover, this work proposes a highly efficient design of the amplification block based on a back-to-back power topology performing a dynamic load modulation exploiting the non-overlapping properties of the de-multiplexed Complex DSM signal. For experimental validation, the proposed de-multiplexed 3-level Delta-Sigma topology was implemented on the BEEcube™ platform followed by the back-to-back Class-E switch-mode power amplification block. The full transceiver is assessed using a 4th-Generation mobile communications standard LTE (Long Term Evolution) standard 1.4 MHz signal with a peak to average power ratio (PAPR) of 8 dB. The dual-branch topology exhibited a good linearity and a coding efficiency of the transmitter chain higher than 72% across the band of frequency from 1.8 GHz to 2.7 GHz.

## 1. Introduction

The increasing demand of the new telecommunication standards in terms of bandwidth, spectral efficiency and power efficiency has led to the development of novel transceiver architectures and a continuous improvement in their performance. In the case of broadband and multi-standard Software Defined Radio (SDR) transmitters, Delta-Sigma Modulator (DSM)-based transmitters have shown a good linearity [1] and a high efficiency [2]. Particularly for transmitters where the requirements of high power efficiency, wide coverage range and autonomy are stringent. This is in addition to the limitations of hardware processing resources, which prevent the implementation of complex linearization techniques.

Previous works have focused on improving the performance of DSM-based transmitters in terms of Coding Efficiency (CE) and improvement of the DSM quantization noise shaping, mainly by increasing the order of the DSM or increasing the number of quantization levels. In [3,4], multi-level DSM was investigated, dual-band DSM transmitters were demonstrated in [5,6] focused on the application of carrier aggregation on DSM signals. 

In this work, a topology based on Complex Delta-Sigma Modulation (CxDSM) is implemented, composed of a baseband signal-processing block, an up-conversion block and, finally, an amplification block. The main feature of the CxDSM is that it takes into account the phase in the feedback loop while the output modulated signal is kept at a discrete level. The baseband block performs a 3-level DSM quantization and then de-multiplexes into two separate streams based on the magnitude of the DSM signal. This topology aims to improve the overall efficiency of the transmitter using an amplification block based on combined and non-isolated Switch-Mode Power Amplifiers (SMPAs) while preserving the signal quality. The measurement results from the hardware implementation are presented and discussed. 

In Section 2, the main theory of the CxDSM, as well as the multi-bit quantization property, are explained. In Section 3, the 3-level de-multiplexed DSM topology is proposed. The implementation steps of the amplification block are presented in Section 4, and the performance evaluation of this solution is presented in Section 5.

## 2. Complex Multi-Bit Delta-Sigma Modulator

**Figure 1:**
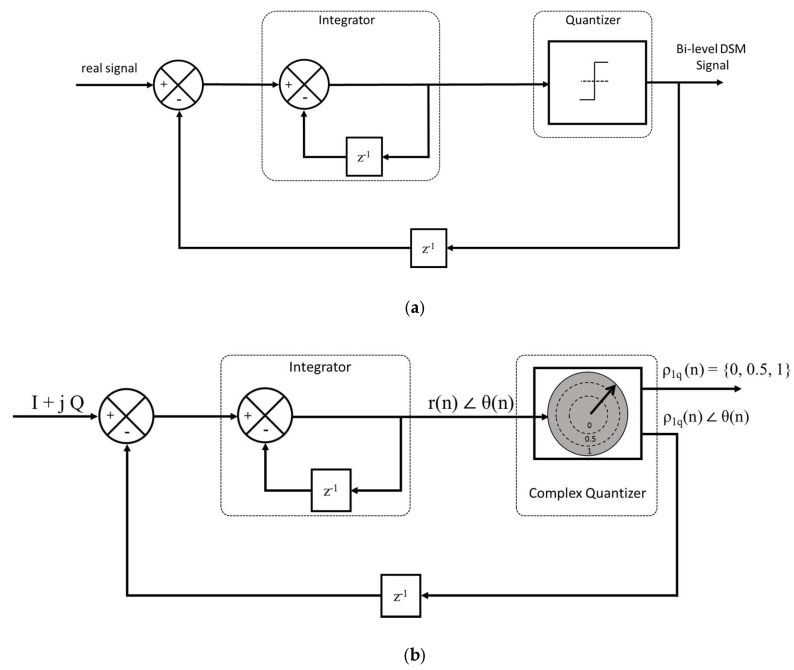
(**a**) Delta Sigma modulator blocks; (**b**) CxDSM block diagram.

The fundamental building blocks of a DSM are a subtractor, an integrator, a quantizer and a feedback loop, as shown in Figure 1a. 

The integrator presents a first-order low-pass transfer function to the input signal. The 1-bit quantizer has an output bit-stream of 0 s (zeros) and 1 s (ones), hence the conversion of a continuous signal into a bi-level modulated signal. This output bi-level signal is suitable for driving a Switching Mode Power Amplifier (SMPA) [6]. The quantizer should operate at a high oversampling frequency. The quantizer compares the module of the signal to the two thresholds and outputs the 3-level signal at the main output. We denote the oversampling ratio (OSR), calculated as the sampling frequency divided by twice the bandwidth; the ratio should be ten times or higher. The OSR is increased to reduce the quantization error. Thus, a better noise shaping is obtained. This out-of-band noise represents the main part of the signal. Hence, a major part of the SMPA power will be dissipated by the amplification of this quantization noise [7]. 

In this work, we used the CxDSM, as it presents a higher coding efficiency and reduces the power of the quantization noise [3]. The CxDSM in Figure 1b was first presented in [8,9], and is based on simultaneous quantization of the in-phase component (I) and the quadrature-phase component (Q) of a complex signal. This technique exhibits more accurate quantization of the signal, resulting in higher coding efficiency and linearity than its Cartesian DSM (CDSM) counterpart [3]. 

The coding efficiency is a typical figure of merit that affects the overall power efficiency of DSM transmitters. It is defined as the ratio of the desired signal power to the total signal power:

(1) $$CE\%=\frac{SignalPower}{SignalPower+Out\;of\;band\;quantization\;noise}\times 100$$

Typical values for coding efficiency reported in two-level LP-CDSMs can be as low as 9% [5]. Such poor performance of the amplifier drastically impacts the overall efficiency of the transmitter [1].

The three-level de-multiplexed Low-Pass Complex Delta-Sigma Modulator (LP-CxDSM) architecture proposed in [3] offers a higher coding efficiency and lower overall power consumption compared to the single branch one-bit DSM. It also prevents the SMPA from being prematurely saturated by the high amount of quantization noise.

Compared with the Low-Pass Cartesian Delta-Sigma Modulator (LP-CDSM), which needs to decompose the signal symmetrically into five levels (−1, −0.5, 0, 0.5 and 1), the LP-CxDSM equivalent needs only three quantization levels (0, 0.5 and 1) for both the I and Q streams. Furthermore, an additional phase quantization of the combined signal is required to further decrease the quantization noise of the CxDSM.

Therefore, demultiplexing the three-level complex quantizer into two bi-level quantizers using two thresholds is adopted.

In fact, increasing the quantization levels in DSM improves the spectral efficiency [7] at the expense of increasing the complexity of the hardware implementation and processing resources; using three levels is an effective trade-off between efficiency and complexity. Moreover, the multi-level DSM output signal is not a constant envelope signal, and this degrades the efficiency of the SMPA.

## 3. Dual-Branch DSM Topology

The proposed architecture mainly comprises a baseband processing block, a frequency up-conversion block, and an amplification block followed by an RF combining network.

**Figure 2:**
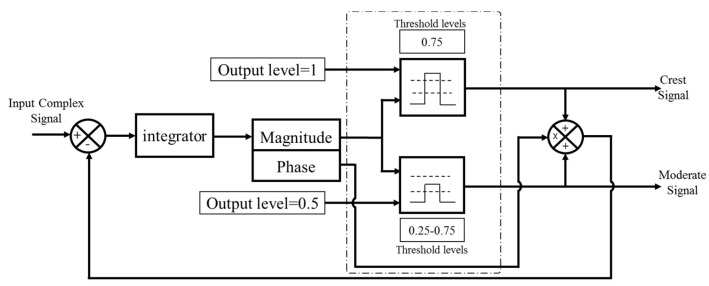
The three-level de-multiplexing quantization process.

All operations, such as oversampling and interpolation, three-level DSM signal generation—shown in Figure 2—and signal shaping are performed within the baseband processing block. The three-level output signal is de-multiplexed into two bi-level signals at the same oversampled frequency, which are output in two separate transmission streams. 

**Figure 3:**
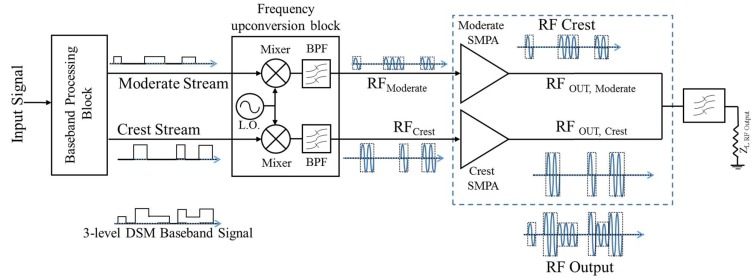
Dual-branch DSM architecture.

The Moderate stream and the Crest stream are generated by two separate transmitters. In order to perform the de-multiplexing process, the three-level DSM circuit is decomposed into two separate quantization circuits, as shown in Figure 3; the Moderate quantizer outputs a signal with a level of a 0.5 whenever the signal lies in between the thresholds 0.25 and 0.75; otherwise, the output is zero. Similarly, the Crest quantizer generates the ones signal every time the signal exceeds the threshold of 0.75; and otherwise, zeros.

In multi-level DSMs, the quantized signal is no longer a constant-envelope signal. Consequently, the SMPA is not driven at its saturation power most of the time, when the quantized signal is not at its maximum value. This will impact the overall efficiency. By generating the two bi-level signals out of the three-level signal, this architecture aims to overcome the efficiency loss and enhance the coding efficiency.

Increasing the number of quantization levels for this topology requires an increase in the number of the quantization thresholds; hence, we end up with a more complex quantizer architecture. This will also require an increase in the number of branches and numbers of the SMPAs, and this would drastically degrade the overall efficiency and linearity of the DSM, and would require higher processing speed and a more sophisticated quantizer design.

The output signal of the DSM circuit is split into two bi-level streams, Crest and Moderate streams. Hence, two constant envelope RF signals are generated, the two baseband streams are frequency up-converted by mixing them with the desired RF carrier frequency. 

After up-conversion around the carrier frequency, each constant envelope RF signal is fed to the respective SMPA working at saturation mode. 

**Figure 4:**
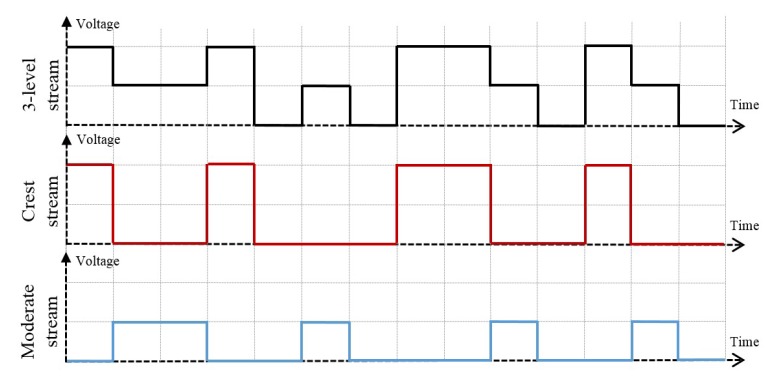
Time-domain decomposition of the three level-signals.

Figure 4 shows the time-domain representation of the output signal of the dual-branch de-multiplexing quantizer. In one scenario, the Crest SMPA is driven at saturation, while the Moderate SMPA is biased and has no RF input signal. In this case, the Crest SMPA is ON and the Moderate SMPA is OFF. In the second scenario, the Moderate SMPA is driven at saturation while the Crest SMPA is biased and has no RF input signal. In this case, the Moderate SMPA is ON and the Crest SMPA is OFF. Otherwise, both SMPAs are OFF and the output is zero.

The de-multiplexed signal properties were taken into consideration while designing the amplification block.

## 4. The Amplification Block Design

A class-E SMPA is used for each branch to build the amplification block. The implemented class-E SMPAs are matched to operate simultaneously in a high-efficiency mode using a solution based on a T-junction combiner.

Instead of using an isolated power combiner such as a Wilkinson power combiner, which has a 3 dB loss, the proposed combiner is designed to maximize the performance of the SMPAs. The 50 Ohm T-junction combines the RF_Crest_ and moderate output signals of the two branches.

Unlike the solution using the H-Bridge to drive the DSM signals in [10], the proposed amplification block doesn’t require an isolated power combiner based on transmission lines and a balun. The non-overlapping time-domain property between RF_Crest_ and RF_Moderate_ was taken into consideration while designing the T-junction combiner and the matching networks for each SMPA, such that the SMPA that is off will show a high output reflection coefficient—namely, an open circuit on the Smith chart—to allow the operating SMPA to output its maximum power. The equivalent circuit to the SMPA when the input signal is zero is a quasi-open switch. This could be achieved by tuning the length of the transmission lines to tune the phase of the reflection coefficient at the operating frequency. The RF T-junction combining network is designed in such a way as not to introduce a loss, while preserving the overall efficiency. Each of the T-junction 50 Ohm line lengths is designed to compensate for the phase rotation. The phase rotation moves the impedance away from the open circuit point on the smith chart. 

**Figure 5:**
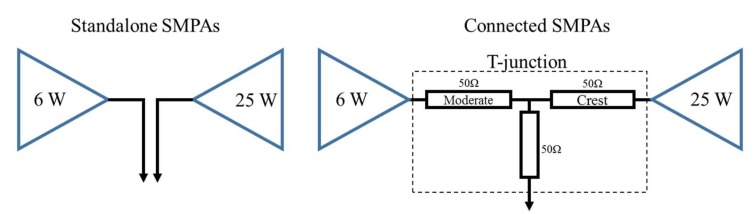
Standalone SMPAs and connected SMPAs and their drain efficiency equations.

The SMPAs are designed with relatively high output reflection coefficients (S22). This reflection coefficient is represented by S22 by the S-parameters on the Smith chart. The length of the T-junction branch is selected to bring the output impedance to the open circuit on the Smith chart. The designed 25 W crest SMPA presented in Figure 5 covers the wideband design frequency from 1.8 GHz to 2.7 GHz and shows a high magnitude for different values throughout the operating frequency band [11].

A degradation in the overall performance takes place due to the power leakage in the OFF SMPA as the value of the reflection coefficient is limited to values (Γ_OUT_OFF_ < 1) throughout the entire operational frequency band. This is in addition to the imbalance between the reflection coefficients of the two branches. 

Similarly, the reflection coefficient S22 of each SMPA, noted as Γ_OUT_OFF_ in Table 1, of the 6 W moderate SPMA and the 25 W SMPA are presented in Table 1, along with the characteristics of each SMPA.

We note that the difference of maximum powers between the Crest and Moderate SMPAs is roughly 6 dB, which is also equal to the double of the magnitude of the factor between the Crest and Moderate signal output voltages.

An amount of output power leaks from the Crest SMPA to the Moderate SMPA. Moreover, when the Crest SMPA is ON, a large amount of power appears at the output terminal of the OFF Moderate SMPA. Hence, more current is more likely to be drawn at the drain terminal of the OFF Moderate SMPA.

The calculated output power and the drain efficiency of the two standalone SMPAs connected with an isolated combiner is higher than the combined SMPAs. However, the efficiency of the combined SMPAs is degraded due to the power leakage to the OFF-SMPA. This limitation cannot be totally avoided, due to the imperfect value of the *Γ_OUT_* when of the SMPAs are Off as the matching point could be away from the open-circuit condition.

The total efficiency of the three-level de-multiplexed DSM-based transmitter (*η_T_*) is given by:

(2) $$\eta_{T}=CE\times ({\bar{P}}_{\mathrm{crest}}\times \eta_{SATCrest}+{\bar{P}}_{\mathrm{Moderate}}\times \eta_{SATModurate})$$

where *CE* denotes the coding efficiency of the three-level DSM, ${\bar{P}}_{\mathrm{crest}}$ is the relative probability of occurrence of the Crest level in the three-level quantized signal before de-multiplexing, and ${\bar{P}}_{\mathrm{moderate}}$ is the relative probability of occurrence of the Moderate level in the three-level quantized signal before de-multiplexing. *η_SATCrest_* and *η_SATModerate_* are, respectively, the efficiencies of Crest SMPA and Moderate SMPA at saturation.

The measured difference between the output powers of the Crest SMPA and the Moderate SMPA is equal to 5.3 dB lower than 6 dB. Thus, the linearity of the transmitter is degraded when driven with a three-level quantized signals with 1, 0.5, and 0 levels.

To remedy these impairments and preserve the linearity of the transmitter, the three-level LP-CxDSM had to be tuned to output a signal that compensates the difference between the output powers of the two SMPAs to reach exactly 6 dB.

## 5. Dual-Branch DSM Implementation

The baseband processing block was implemented on the BEECube™ platform. A BEECube™ platform is composed of a Virtex 6 FPGA and computer mother board. The platform is equipped with an FMC board, a 2-channel transmitter and 2-channel receiver front-end.

The platform was used to generate the dual-branch DSM signal. The building blocks of the DSM were implemented on the Xilinx™ SysGen™ and MATLAB^®^ running on the BEECube™. 

The BEEcube™ front-end board showed an imbalance between the two paths. Hence, a correction was applied to overcome this imbalance. Furthermore, magnitude and phase correction factors were included in the baseband processing block to compensate for non-linearity and hardware impairments such as I/Q imbalance and DC offset. 

Better linearity of the transmitter chain is achieved by adjusting the levels of the de-multiplexed output signals at the output of the baseband processing according to the output power of the SMPAs throughout the frequency band. The measured levels of the output signal for the Crest signal vary from 0.86 to 1.

**Figure 6:**
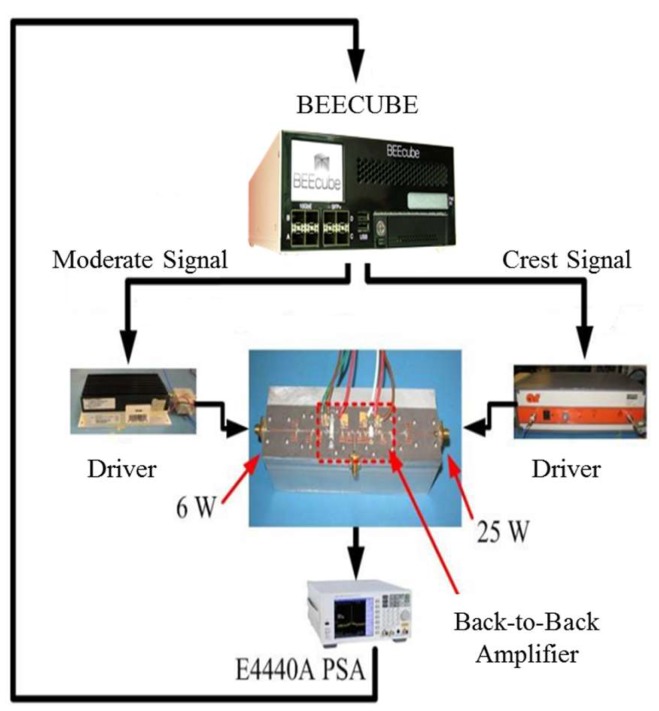
Dual-branch DSM setup.

The setup of the de-multiplexed DSM shown in Figure 6 is composed of the BEEcube™, the driver PAs, the amplification block, and the spectrum analyzer. A triggering signal is needed between the BEEcube™ and the PSA spectrum analyzer.

## 6. Dual-Branch DSM Performance

The oversampled signal bandwidth processed by the platform must be relatively narrow compared to the speed of the processing clock. This is due to the required high oversampling ratio being limited by the sampling speed of the Digital to Analog Converters (DAC) on the SDR platform. The processing speed on the FPGA presents another limitation for the bandwidth. Unbalanced branches can cause the samples to overlap in the time domain. Similar-length cables were used to ensure that the effective length of the two branches was equal, in addition to a static compensation block for time offset and phase rotation, which was implemented in the baseband processing unit of one of the branches. Increasing the bandwidth of the signal will make the alignment between the two branches more critical in preventing the two-branch inter-symbol overlapping. Thus, a high oversampling ratio should be considered to avoid the time mismatch when combining the two branches. 

A high sampling speed is required by the CxDSM to reach the high OSR necessary for good noise shaping. This constraint presents the main limitation to the signal bandwidth at the input of DSM. On the BEECube, the TX sampling speed is 245.76 MS/s. To overcome this limitation, in-band noise filtering of the Quantization Noise Reduction QNR with In-band Filtering (QNRIF) [3] is applied at the output of the DSM. The baseband processing block will split the 3-level signal into two 2-level streams, then the QNRIF is applied to each stream separately. This proposed technique, besides removing a part of the in-band quantization noise, also filters out the out-of-band quantization noise. Therefore, the QNRIF technique will also alleviate the stringent requirement on the bandwidth and its effect on the adjacent channels. Consequently, the improved ACPR at the output of the amplification block can help to ease the constraints on the band-pass filter, leading to a lower insertion loss for the filter and a better overall efficiency for the transmitter. Consequently, an enhanced efficiency and signal-to-noise ratio can be achieved simultaneously for wideband signals. Although this technique improves the ACPR significantly, the bi-level signals are disturbed and will introduce some ripples in the constant levels of the bi-level signals. 

**Figure 7:**
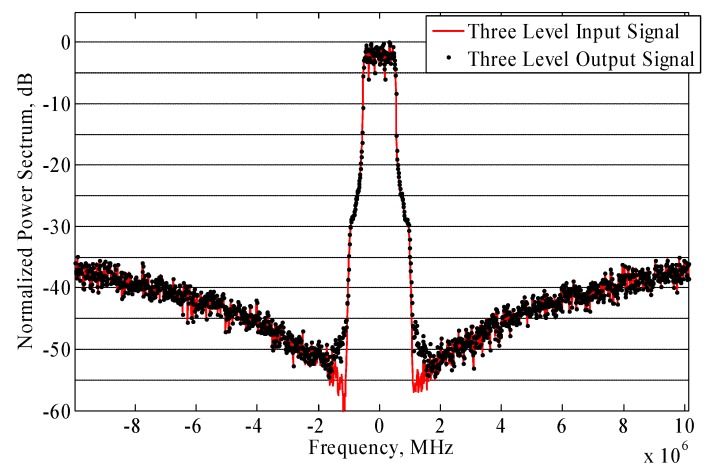
Normalized input and output three-level CxDSM signal.

**Figure 8:**
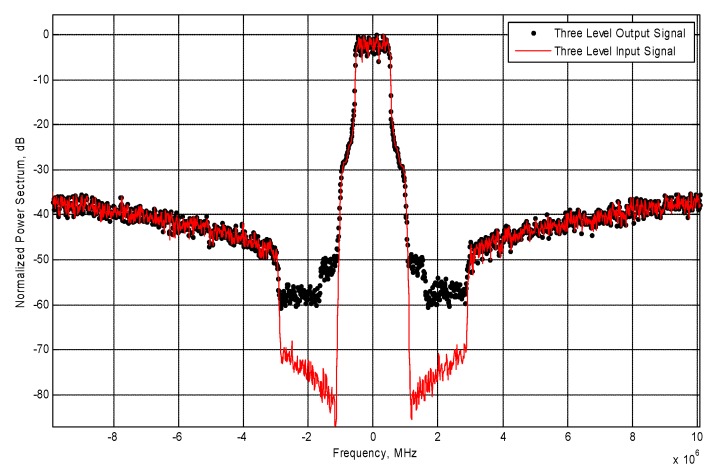
Normalized input and output three-level DSM signal after QNRIF.

The application of the QNRIF to the output signal of the DSMs attenuates the power in the adjacent channel. The QNRIF technique effect on the adjacent channel is depicted in Figure 7 and Figure 8.

The use of the QNRIF technique significantly improves the Adjacent Channel Power Ratio (ACPR) measured at 1.4 MHz offset. The spectrum of the QNRIF output signal is shown in Figure 8.

**Figure 9:**
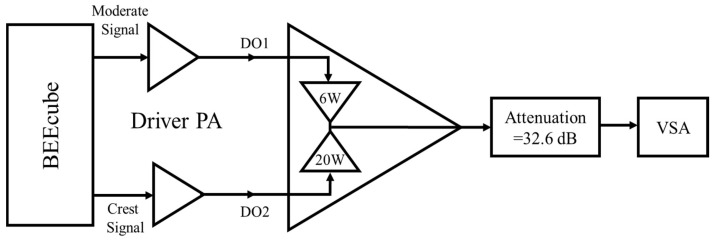
The amplification block stage setup.

The designed amplification block with two inputs and one output to amplify and combine the two signals was tested according to the setup in Figure 9, we used a driver power amplifier for each branch, and we tested the block under different conditions. Both the SMPAs were biased and tested separately; one SMPA was under test, while the other SMPA was matched. Ideally, the combined amplifiers have an output reflection coefficient magnitude equal to 1, the measured reflection coefficient for the OFF SMPAs were 0.82 and 0.88 for the 6 W SMPA and 25 W SMPA, respectively. Afterwards, both SMPAs were tested simultaneously. A 32.6 dB attenuator was used after the amplification block.

Starting with a continuous-wave (CW) test, the biasing voltages are: V_gs_ = −4 V, V_gs_ = −5 V for the 25 W and 6 W SMPA respectively at a carrier frequency of f_c_ = 2.1 GHz with PDO1 and PDO2 denoting respectively the output power of the driver 1 and 2. The CW test results are illustrated in Table 2.

The performance of the proposed transmitter is evaluated using a LTE signal having a bandwidth of 1.25 MHz and 8dB PAPR. *V_gs_*(25 W) = −4 V, *V_gs_*(6 W) = −5 V, Freq = 2.1 GHz, PAPR(0.5’s) = 3.6 dB, PAPR(1’s) = 12.4 dB. The obtained results are presented in Table 3 below.

Using the 1.25 MHz LTE signal with both SMPAs excited. We evaluate the in-band and the total band specification of the signal at a frequency of 2.1 GHz. The measured results are specified in the Table 4, for the same configuration: Vgs(25 W) = −4 V, Vgs(6 W) = −5 V, Freq = 2.1 GHz, PAPR(0.5’s) = 3.6 dB, PAPR(1’s) = 12.4 dB.

A frequency sweep across the total bandwidth from 1.8 GHZ to 2.7 GHz covered by the transmitter shows the flatness of frequency response and the inherent linearity of the transmitter as shown in Table 5.

The quantization levels were tuned to further improve the linearity. The Normalized Mean Square Error (NMSE) after linearization of the BEEcube™ dual-channel transmitter reached the value of −43.35 dB.

The transmitter has a maximum measured power efficiency of 43.9 %, an output SNDR above 40 dB and a measured ACPR of −45 dB for a signal with PAPR= 12.2 dB. 

The peak output power of the amplification block at each carrier frequency of the three-level first-order de-multiplexed LP-CxDSM is tuned to preserve a flat response across the whole band. A considerable improvement was achieved by the dual-branch topology in terms of performance compared with the single-branch 3-level First-Order DSM, with an oversampling ratio of 16 with performances in SNDR = 20 dB and a Coding Efficiency of 28.8%. 

## 7. Conclusions

In this work, we proposed a novel two-branch transmitter topology using a three-level complex DS modulation signal encoding scheme and two switching mode amplifiers connected using a non-isolated power combiner. A prototype was designed, implemented using SDR platform and tested to assess its performance. An SNDR of 46 dB was achieved by splitting the three-level signal into two bi-level constant-envelope signals. We used a non-isolated T-junction combiner to improve the transmitter efficiency. The proposed architecture is inherently linear and does not require a further linearization processing.

By integrating a quantization noise reduction technique in the baseband processing block, we enhanced the ACPR considerably and reached a coding efficiency higher than 75%. This topology, along with the proposed signal processing techniques, showed a good overall performance.

## Figures and Tables

**Table:** 

$\eta_{Drain}=\frac{P_{OUT\_ON}}{V_{DS\_ON}\times I_{DS\_ON}}$	$\eta_{Drain}=\frac{P_{OUT\_ON}}{\left(V_{DS\_ON}\times I_{DS\_ON})+(V_{DS\_OFF}\;\times I_{DS\_OFF}\right)}$

**Table 1:** SMPA specifications.

	Moderate SMPA	Crest SMPA
Device	6 W CGH40006 from Cree Inc.	25 W CGH40025 from Cree Inc.
*P_OutMax_* (dBm)	36.7	42.2
*η_Drain_* (%)	67	52
*Γ_OUTOFF_*	0.82	0.88

**Table 2:** Continuous wave tests.

Scenario:1 6 W Under Test, 25 W Terminated		Scenario 2: 6 W Terminated, 25 W Under Test	
PDO1 (dBm)	26	PDO2 (dBm)	34
Pout (dBm)	36.2	Pout (dBm)	41.8
Efficiency %	69.9	Efficiency %	36.8

**Table 3:** LTE modulated signal tests.

Scenario 1: 6 W Under Test, 25 W Terminated			Scenario 2: 6 W Terminated, 25 W Under Test		
PDO1 (dBm)	peak	25.8	PDO2 (dBm)	peak	35.4
	average	22.2		average	23
Pout (dBm)	peak	35.9	Pout (dBm)	peak	41.7
	average	32.3		average	29.3
Efficiency %	55.5		Efficiency %	30.5	

**Table 4:** Performances for 2.1 GHz frequency.

	Whole Band	In Band
Peak Power (dBm)	41	39.3
Average Power (dBm)	34.9	33
Efficiency %	43.9	30.7
Coding Efficiency %	76.8	

**Table 5:** Transmitter performances over the covered bandwidth.

Carrier Frequency (GHz)	Measured CE%	Pout Average (dB)	Transmitter Efficiency %	Output SNDR (dB)
1.8	74.6	34.1	34	40
1.9	75	34.7	35.5	41
2	76	35.1	38.4	42
2.1	76.8	34.9	43.9	43
2.2	75.1	34.8	42.9	44
2.3	75	34.4	39.1	45
2.4	73.4	33.2	38.9	42
2.5	74.3	32.2	35.9	43
2.6	72.4	32.6	42.3	44
2.7	72.6	33.1	42.5	43
